# Tumor Lysis Syndrome Rare Presentation As Uremic Pericarditis: A Case Report

**DOI:** 10.7759/cureus.35727

**Published:** 2023-03-03

**Authors:** Fábio Caleça Emidio, Rafaela Pereira, Pedro Martins dos Santos, Teresa Abegão

**Affiliations:** 1 Internal Medicine Department/Internal Medicine, Centro Hospitalar Universitário do Algarve - Hospital de Faro, Faro, PRT

**Keywords:** water-electrolyte imbalance, acute kidney injury, pericarditis, chemotherapy, colonic neoplasms, tumor lysis syndrome

## Abstract

Tumor lysis syndrome (TLS) is an oncological emergency characterized by the massive destruction of malignant cells and the release of their contents into the extracellular space, which might occur spontaneously or post-chemotherapy. According to the *Cairo&Bishop Classification*, it can be defined by both laboratory criteria: hyperuricemia, hyperkalemia, hyperphosphatemia, hypocalcemia (two or more); and clinical criteria: acute kidney injury (AKI), convulsions, arrhythmias, or death. We report the case of a 63-year-old man with a previous medical history of colorectal carcinoma and associated multiorgan metastasis. The patient was initially admitted to the Coronary Intensive Care Unit, five days after the chemotherapy session, on suspicion of Acute Myocardial Infarction. Upon admission, he presented without significant elevation of myocardial injury markers, but with laboratory abnormalities (hyperkalemia, hyperphosphatemia, hyperuricemia, and hypocalcemia) and clinical symptoms (sudden sharp chest pain with pleuritic characteristics and electrocardiographic anomalies suggesting uremic pericarditis, and acute kidney injury), all consistent with TLS. The best approach to established TLS is aggressive fluid therapy and a decrease in uric acid levels. Rasburicase proved to be notoriously more effective, both in terms of prevention and treatment of established TLS, thus consisting of the first-line drug. However, in the present case, rasburicase was not available at the hospital level, so a decision was made to initiate treatment with allopurinol. The case evolved with slow but good clinical evolution. Its uniqueness resides in its initial presentation as uremic pericarditis, scarcely described in the literature. The constellation of metabolic alterations from this syndrome translates into a spectrum of clinical manifestations that can go unnoticed and ultimately may prove to be fatal. Its recognition and prevention are crucial for improving patient outcomes.

## Introduction

Tumor lysis syndrome (TLS) is an oncological emergency in which there is massive destruction of malignant cells and release of their contents into the extracellular space, either spontaneously or after chemotherapy [1]. Associated, in most cases, with hematological malignancies with a high proliferative index - namely acute lymphoblastic leukemia and high-grade non-Hodgkin's lymphomas - more recently, it has been described with greater frequency, even in the context of solid neoplasms [2]. This increment in reported incidence probably reflects the increased recognition of this condition, and the use of newer, more effective cytotoxic drugs and those used in combined regimens. According to the *Cairo&Bishop Classification*, it is defined by the presence of two or more laboratory criteria (hyperuricemia, hyperkalemia, hyperphosphatemia, and hypocalcemia), in the period between three days before and seven days after the start of chemotherapy; and clinical criteria: worsening renal function (AKI), convulsions, arrhythmias or death [3]. This definition has several limitations: it implies the initiation of chemotherapy, not considering situations of spontaneous TLS, uses a serum creatinine value greater than 1.5 times the normal as a diagnostic criterion for AKI, and does not mention the need for simultaneous occurring metabolic changes [4,5]. However, it remains the most used classification. Due to the reserved prognosis with which it is associated, the prevention of its occurrence per se, as well as its consequences, is mandatory [1,6].

## Case presentation

We present the case of a 63-year-old man with a medical history of colon adenocarcinoma diagnosed in December 2016, who underwent left hemicolectomy and adjuvant therapy. In February 2019, a positron emission tomography (PET) scan was performed, revealing a hypermetabolic lesion of the sternal notch, with soft tissue involvement; right pulmonary hilar lesion; hepatic parenchyma (segments V, VI, and VII) and a subcentimeter abdominal hypermetabolic nodule, near the head of the pancreas. At this time, he starts a chemotherapy regimen with *FOLFOX* (5-fluorouracil/folinic acid with oxaliplatin) plus bevacizumab, which he kept up to the date of admission. The patient was initially admitted to the Coronary Intensive Care Unit (CICU) due to suspicion of acute myocardial infarction. Upon admission he complained of sudden chest pain over the anterior chest, beginning 24 hours before, accompanied by hypersudoresis. The pain was described as sharp, exacerbated by inspiration and coughing, but with an intensity decrease, when sitting up and leaning forward. At physical examination, the patient was distressed, although hemodynamically stable, but with no alterations upon cardiac and pulmonary auscultation or other physical findings. He also complained of worsened asthenia and anorexia, since the last chemotherapy session (five days before the onset of the symptoms). The study done at the CICU included several electrocardiograms, all showing sinus rhythm, ST-segment elevation with diffuse superior concavity, and PR segment depression, with reciprocal ST segment depression and PR segment elevation in aVR and V1 derivations. A transthoracic echocardiogram was also performed, showing only a slight pericardial effusion, with no altered valvular or cardiac function. Laboratory tests revealed Troponins < 100 pg/mL (normal male cut-off 34.2 pg/mL); worsening Creatinine (increase from 1.0 to 5.0 mg/dL, in five days - N: 0.7-1.3 mg/dL); blood urea nitrogen (BUN): 102 mg/dL (N: 8.4-25.7 mg/dL); mild hyperkalemia: 4.6 mg/dL (N: 3.6-5.1 mmol/L); hyperuricemia: 12 mg/dL (N: 3.5-7.2 mg/dL); hypocalcemia/corrected calcium level for hypoalbuminemia of 8.7 mg/dL (N: 9-10 mg/dL) and hyperphosphatemia: 7.6 mg/dL (N: 2.3-4.7 mg/dL). Although these findings were suggestive of acute pericarditis, a diagnosis of low-risk non-ST-elevation myocardial infarction (NSTEMI) was not completely discarded up to this point. Thus, dual antiplatelet therapy with ticagrelor and acetylsalicylic acid was instituted, and catheterization was postponed. On the following day, due to clinical stabilization (stable chest pain) and no significant increase in markers of myocardial injury, acute cardiac infarction was discarded, and a diagnosis of pericarditis was assumed. However, there was some difficulty with the interpretation and correlation of both clinical and laboratory findings, so collaboration was requested with the Intermediate Care Unit (IMCU)/Internal Medicine. Based on both laboratory findings (hyperkalemia, hyperphosphatemia, hyperuricemia, hypocalcemia), and clinical findings (sudden sharp chest pain plus pericardial effusion, probably secondary to Uremic Pericarditis) and AKI, the diagnosis of TLS was assumed. The patient was then transferred to IMCU, where vigorous intravenous hydration was instituted (saline solution 4L per day), furosemide 20 mg every four hours, and allopurinol 100 mg every 12 hours (due to the unavailability of rasburicase at the hospital). To exclude AKI of obstructive etiology, a renal ultrasound was requested, which did not show signs suggestive of obstruction and/or other relevant findings.

## Discussion

In the reported case, the patient is initially admitted to the CICU on suspicion of acute coronary syndrome, later excluded. However, based on clinical and electrocardiographic findings, the probable cause of admission was uremic pericarditis. According to *Cairo&Bishop*, two laboratory criteria were met (hyperphosphatemia and hyperuricemia). Although mild hypocalcemia and hyperkalemia were also present, they were not considered as fulfilled criteria since the pre-defined cut-off was not met. A clinical criterion (AKI) was also fulfilled, five days after chemotherapy (the close temporal association constitutes a strong predictor for this syndrome). Clinically, neither arrhythmia nor convulsive crises coexisted, and only cardiac involvement with chest pain was observed, presumably secondary to uremic pericarditis (Table 1) [3].

**Table 1:** Laboratory and clinical criteria for tumor lysis syndrome according to Cairo&Bishop criteria *Three days before to seven days post-chemotherapy

Laboratory Criteria*	
Hyperkalemia	4.5mg/dl ( > 6 mEq/L or > 25% baseline) - Not fulfilled
Hyperphosphatemia	7.6mg/dl ( > 4.5 mg/dl or > 25% baseline) - Fulfilled
Hyperuricemia	12mg/dl ( > 8 mg/dl or > 25% baseline) - Fulfilled
Hypocalcemia	Corrected with albumin 8.7mg/dl ( < 7 mg/dl or < 25% baseline) - Not fulfilled
Clinical Criteria	
Acute Kidney Injury (AKI)	Creatinine increase from 1.0 to 5.0 mg/dl in 5 days Post-Chemotherapy ( > 1.5x Upper normal limit) - Fulfilled
Arrythmia	Not fulfilled
Convulsions	Not fulfilled

The exclusion of pre-renal injury, nephrotoxic agents, and obstruction were supportive of intrinsic renal injury, probably occurring through a mechanism of intratubular precipitation of uric acid and calcium phosphate crystals, resulting from massive tumor lysis. The best approach to established TLS is aggressive fluid therapy and a decrease in uric acid levels. Rasburicase, a recombinant urate oxidase, leads to the degradation of uric acid into allantoin - a metabolite 10 times more soluble than uric acid. In direct comparison with allopurinol, rasburicase proved to be notoriously more effective, both in terms of prevention and in the treatment of established TLS, thus consisting of the first-line drug [7,8]. However, in the present case, rasburicase was not available at the hospital level, so a decision was made to initiate treatment with allopurinol, besides its several limitations. Allopurinol acts by decreasing uric acid formation, so does not reduce the preexisting serum uric acid. It might increase serum levels of the purine precursors hypoxanthine and xanthine, which may lead to xanthinuria, deposition of xanthine crystals in the renal tubules, and aggravation of AKI. Concomitantly, in the face of worsening AKI and severe hydro electrolytic alterations refractory to medical therapy, it may be necessary to institute a renal replacement technique (RRT) [5]. RRT was considered, but ultimately not necessary in the present case, since the patient improved with increased diuresis and slowly reestablished kidney function. The potential for rapid deterioration in these patients and the need for close surveillance (including dysrhythmic changes) requires monitoring in a differentiated unit. The patient remained under surveillance at the IMCU for eight days. During this period, he experienced a slow but good clinical evolution. At the date of transference to the oncology ward, he presented with a creatinine value of 3.6 mg/dL, diuresis above 50cc per hour, with water-electrolytic imbalance resolution.

## Conclusions

TLS is an important complication of chemotherapy and malignancies with a high proliferative index. Classically associated with hematological malignancies (leukemia/lymphomas), given the larger tumor mass and proliferative capacity, the potential for cell lysis must be considered in line with increasingly effective chemotherapy modalities. Therefore, the incidence of TLS is increasingly reported in neoplasms with which it was classically not associated. The best approach to established TLS is aggressive fluid therapy and a decrease in uric acid levels. Rasburicase is a first-line drug in the prevention and treatment of established TLS. However, in the present case, rasburicase was not available, so a decision was made to initiate treatment with allopurinol, which resulted in clinical improvement. Its uniqueness resides in its initial presentation as uremic pericarditis, scarcely described in the literature. The constellation of metabolic alterations from this syndrome translates into a spectrum of clinical manifestations that can go unnoticed and ultimately may prove to be fatal. Its recognition and prevention are crucial for improving patient outcomes.

## References

[1] Howard SC, Jones DP, Pui CH (2011). The tumor lysis syndrome. N Engl J Med.

[2] Wilson FP, Berns JS (2014). Tumor lysis syndrome: new challenges and recent advances. Adv Chronic Kidney Dis.

[3] Cairo MS, Coiffier B, Reiter A, Younes A (2010). Recommendations for the evaluation of risk and prophylaxis of tumour lysis syndrome (TLS) in adults and children with malignant diseases: an expert TLS panel consensus. Br J Haematol.

[4] Cairo MS, Bishop M (2004). Tumour lysis syndrome: new therapeutic strategies and classification. Br J Haematol.

[5] Ñamendys-Silva SA, Arredondo-Armenta JM, Plata-Menchaca EP, Guevara-García H, García-Guillén FJ, Rivero-Sigarroa E, Herrera-Gómez A (2015). Tumor lysis syndrome in the emergency department: challenges and solutions. Open Access Emerg Med.

[6] Darmon M, Guichard I, Vincent F, Schlemmer B, Azoulay E (2010). Prognostic significance of acute renal injury in acute tumor lysis syndrome. Leuk Lymphoma.

[7] Feusner J, Farber MS (2001). Role of intravenous allopurinol in the management of acute tumor lysis syndrome. Semin Oncol.

[8] Goldman SC, Holcenberg JS, Finklestein JZ (2001). A randomized comparison between rasburicase and allopurinol in children with lymphoma or leukemia at high risk for tumor lysis. Blood.

